# Nutrition, thyroid function, and tuberculosis: exploring the endocrine–immune axis

**DOI:** 10.3389/ftubr.2026.1945832

**Published:** 2026-10-08

**Authors:** Nooneh Khachatourian, Rubina Takhmazian, Vishwanath Venketaraman

**Affiliations:** Department of Biomedical Sciences, College of Osteopathic Medicine of the Pacific, Western University of Health Sciences, Pomona, CA, United States

**Keywords:** endocrine–immune crosstalk, host susceptibility, hypothyroidism, immune regulation, micronutrients, nutrition, thyroid dysfunction, tuberculosis

## Abstract

Tuberculosis (TB) remains a major global health challenge, with undernutrition recognized as one of the most significant modifiable risk factors influencing disease susceptibility and clinical outcomes. Emerging evidence suggests that nutritional status may also affect TB risk indirectly through its impact on thyroid function and immune regulation. This review examines the current evidence linking nutrition, thyroid dysfunction, and susceptibility to *Mycobacterium tuberculosis* (*M. tuberculosis*), with particular emphasis on the biological mechanisms that connect these factors. Essential micronutrients, including iodine, selenium, iron, zinc, and vitamin D, are critical for thyroid hormone synthesis, metabolism, antioxidant defense, and immune function. Deficiencies in these nutrients can impair thyroid physiology, disrupt endocrine homeostasis, and alter innate and adaptive immune responses that are essential for controlling *M. tuberculosis* infection. Thyroid hormones regulate macrophage activation, T-cell differentiation, cytokine production, immunometabolism, and oxidative stress, suggesting that thyroid dysfunction may compromise host resistance to TB. Conversely, chronic TB infection and antitubercular therapy may further disrupt thyroid function, creating a bidirectional relationship between these conditions. Collectively, the available evidence supports a conceptual framework in which nutritional deficiencies contribute to thyroid dysfunction, thyroid dysfunction impairs antimicrobial immunity, and TB further exacerbates nutritional and endocrine disturbances, establishing a self-perpetuating cycle that may increase disease susceptibility and worsen clinical outcomes. Although direct clinical evidence linking thyroid disorders with TB risk remains limited, the convergence of nutritional, endocrine, and immunological pathways provides a strong biological rationale for further investigation. Future prospective studies are needed to determine whether nutritional optimization, targeted micronutrient supplementation, and early identification and management of thyroid dysfunction can improve immune competence, reduce TB susceptibility, and enhance treatment outcomes in high-risk populations.

## 1 Introduction

Tuberculosis (TB), caused by *M. tuberculosis*, remains one of the leading infectious causes of morbidity and mortality worldwide. Recent estimates indicate that approximately 10.6 million individuals develop TB annually, highlighting the continued global burden of this disease despite ongoing prevention and control efforts (1, 2). Although exposure to *M. tuberculosis* is necessary for infection, progression to active disease is strongly influenced by host-related factors, including nutritional status, immune competence, comorbid conditions, and socioeconomic determinants (2, 3). The ability of the host immune system to contain *M. tuberculosis* is critical, as only a proportion of infected individuals ultimately develop active disease, emphasizing the importance of factors that influence immune function and disease susceptibility (1).

Among these factors, undernutrition has emerged as one of the most important and potentially modifiable risk factors for TB. More than one-fifth of global TB cases are estimated to be attributable to undernutrition, particularly in high-burden regions where food insecurity remains prevalent (4). Systematic reviews have demonstrated that undernutrition significantly increases the risk of developing active TB, while malnutrition is also associated with unfavorable treatment outcomes, including treatment failure, loss to follow-up, and mortality (5, 6). Furthermore, TB itself can contribute to nutritional deterioration through chronic inflammation, increased metabolic demands, reduced appetite, and impaired nutrient utilization, creating a bidirectional relationship between nutritional status and disease progression (6). Consequently, nutritional assessment and intervention are increasingly recognized as important components of comprehensive TB management (4, 6). Beyond its direct role in immune function, nutritional status also influences endocrine pathways that regulate metabolism and host immunity. Among these, thyroid hormones have emerged as important modulators of immune responses and may represent one mechanism through which nutritional status affects susceptibility to infectious diseases such as TB.

Thyroid hormones are essential regulators of metabolism, growth, development, and energy homeostasis. Beyond their well-established metabolic functions, thyroid hormones also influence both innate and adaptive immunity through effects on macrophage activation, cytokine production, oxidative stress regulation, and T-cell responses (7). These immunomodulatory properties suggest that thyroid dysfunction may alter host susceptibility to infectious diseases. Hypothyroidism, one of the most common endocrine disorders worldwide, is associated with metabolic disturbances, chronic low-grade inflammation, and alterations in immune regulation that may impair antimicrobial defense mechanisms (8). As interest in immunometabolism continues to grow, increasing attention has been directed toward understanding how endocrine disorders influence susceptibility to infectious diseases.

Appropriate nutrition plays a critical role in maintaining normal thyroid function. Micronutrients including iodine, selenium, zinc, iron, vitamin A, and vitamin D are required for thyroid hormone synthesis, metabolism, and signaling (7, 9). Deficiencies in these nutrients have been associated with impaired thyroid function, altered immune responses, and increased oxidative stress. In addition, dietary patterns may influence thyroid health through modulation of gut microbiota composition, inflammatory pathways, and nutrient bioavailability, highlighting the complex relationship between nutrition and endocrine function (7). Given that both nutritional deficiencies and thyroid dysfunction can independently affect immune competence, it is plausible that they may collectively influence susceptibility to TB.

In a nationwide population-based cohort study, individuals with hypothyroidism demonstrated an increased risk of subsequently developing TB, while patients with TB also had an elevated risk of subsequent hypothyroidism (10). Although these findings support a bidirectional epidemiological association, the observational study design does not establish causality or determine the biological mechanisms underlying the relationship. Both hypothyroidism and TB are strongly influenced by nutritional status, raising the possibility that micronutrient deficiencies may serve as a common biological factor linking endocrine dysfunction and susceptibility to infection. This concept provides a framework for understanding how nutritional, endocrine, and immunological pathways may converge to influence TB risk. Although the mechanisms underlying this association remain incompletely understood, nutritional status may represent an important biological link connecting thyroid dysfunction, immune dysregulation, and TB susceptibility.

Therefore, the purpose of this review is to examine the current evidence linking thyroid disorders, nutritional status, and susceptibility to TB. Particular emphasis is placed on the immunological and metabolic pathways through which nutrition may influence thyroid function and, consequently, host defense against *M. tuberculosis*. A better understanding of these interactions may provide insight into novel nutritional and therapeutic approaches aimed at reducing TB risk and improving clinical outcomes. The proposed relationship among nutritional deficiencies, thyroid dysfunction, immune dysregulation, and TB susceptibility is illustrated in Figure 1.

**Figure 1:**
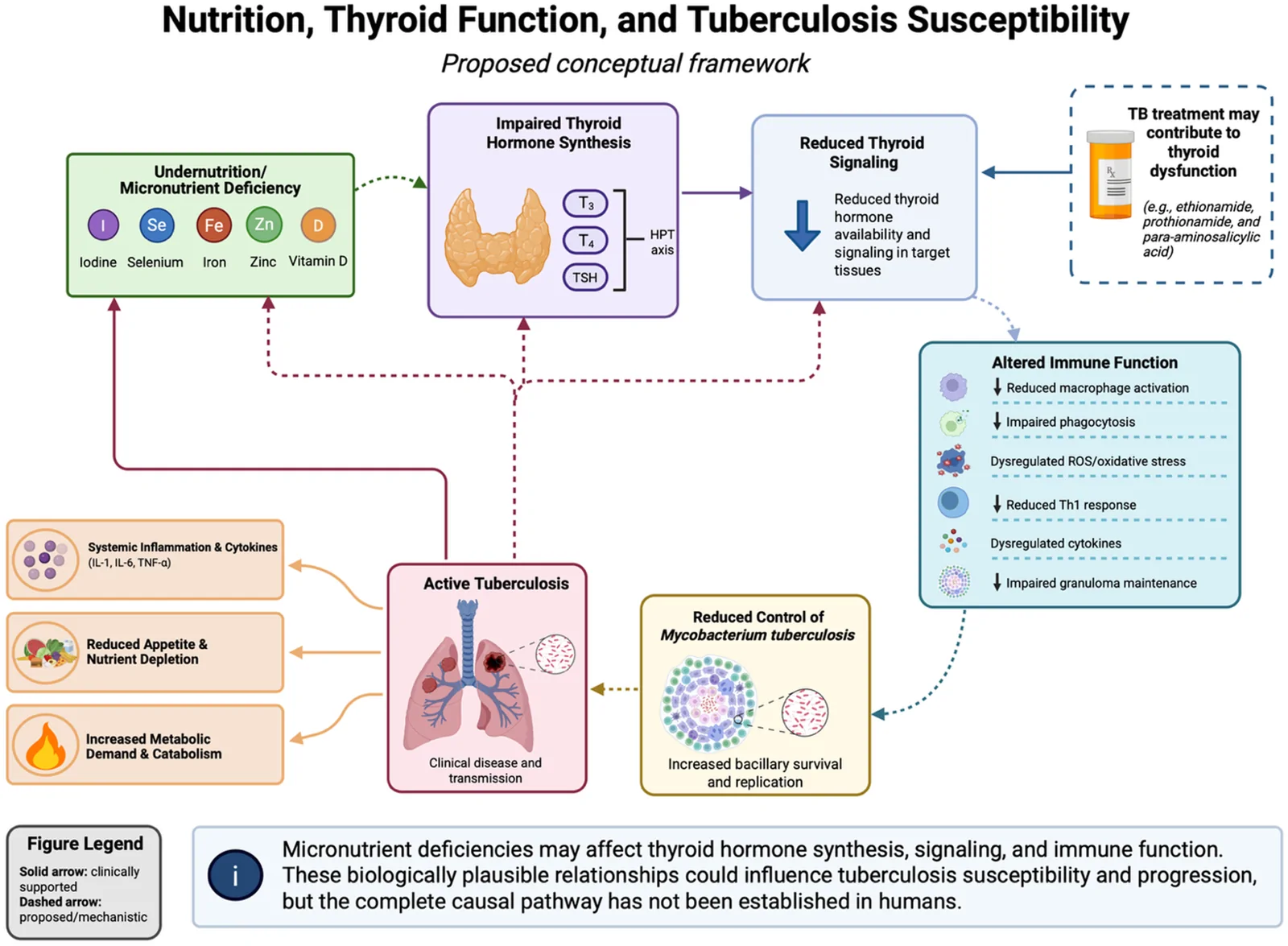
Nutrition, thyroid function, and tuberculosis susceptibility. Proposed conceptual framework illustrating potential relationships among micronutrient deficiencies, thyroid dysfunction, impaired immune responses, and susceptibility to tuberculosis. Solid arrows indicate better established biological, clinical, or epidemiological relationships, whereass dashed arrows indicate proposed or indirect relationships supported primarily by observational or mechanistic evidence. The complete framework should not be interpreted as an established causal pathways. Created in https://BioRender.com.

Throughout this review, evidence is interpreted according to its level and type. Established evidence refers primarily to findings supported by human epidemiological or clinical studies, whereas associative evidence reflects observed relationships that do not establish causality. Mechanistic evidence, including experimental and immunological studies, is used to identify biologically plausible pathways and generate hypotheses regarding potential links among nutritional status, thyroid dysfunction, and TB susceptibility. Given the limited direct clinical evidence connecting thyroid dysfunction with TB risk, proposed endocrine–immune mechanisms should be interpreted as hypothesis-generating rather than causal.

### 1.1 Literature search strategy

A narrative literature search was conducted using PubMed to identify studies examining the relationships among nutritional status, thyroid function, immune regulation, and tuberculosis susceptibility. Searches used combinations of terms including “tuberculosis,” “Mycobacterium tuberculosis,” “thyroid dysfunction,” “hypothyroidism,” “thyroid hormones,” “nutrition,” “micronutrients,” “vitamin D,” “selenium,” “zinc,” “iron,” “iodine,” “immune function,” and “endocrine–immune interactions.” Emphasis was placed on recent peer-reviewed literature published between 2021 and 2026, although older studies were included when relevant to the scope of the review. Priority was given to human studies, systematic reviews, meta-analyses, and recent mechanistic studies relevant to the proposed nutritional–endocrine–immune framework. Studies were included if they addressed one or more of the major relationships examined in this review, including nutrition and thyroid function, thyroid function and immune regulation, nutrition and tuberculosis, or thyroid dysfunction and tuberculosis susceptibility. Studies that were not directly relevant to these relationships or were outside the scope of the review were excluded.

### 1.2 Thyroid disorders and function

Thyroid disorders comprise a diverse group of endocrine conditions characterized by abnormalities in thyroid hormone production, secretion, or action. These disorders may manifest as hypothyroidism, hyperthyroidism, or euthyroid autoimmune disease and can arise from congenital, autoimmune, inflammatory, iatrogenic, or nutritional causes. Among these conditions, hypothyroidism is one of the most prevalent endocrine disorders worldwide and represents a significant public health concern due to its widespread effects on metabolism, cardiovascular health, and immune function. Primary hypothyroidism is characterized by elevated serum thyroid-stimulating hormone (TSH) concentrations accompanied by reduced free thyroxine (fT4) levels, whereas subclinical hypothyroidism is defined by elevated TSH levels despite normal circulating thyroid hormone concentrations (8, 11).

Thyroid hormones play a fundamental role in regulating metabolic homeostasis and influence the function of virtually every organ system. Through interactions with thyroid hormone receptors, triiodothyronine (T3) and thyroxine (T4) regulate energy expenditure, lipid metabolism, carbohydrate utilization, insulin sensitivity, thermogenesis, and body weight maintenance. Thyroid hormones also influence mitochondrial activity and cellular oxygen consumption, thereby affecting overall metabolic efficiency (12). Consequently, thyroid hormone deficiency is associated with reduced metabolic rate, altered lipid profiles, impaired glucose metabolism, increased oxidative stress, and chronic low-grade inflammation (11, 12). These metabolic disturbances may contribute not only to cardiovascular disease and metabolic dysfunction but also to impaired immune competence.

Beyond their classical metabolic functions, thyroid hormones are increasingly recognized as important regulators of immune homeostasis. Emerging evidence demonstrates extensive interplay between the endocrine and immune systems, with thyroid hormones influencing both innate and adaptive immune responses (12, 13). Immune cells including neutrophils, macrophages, dendritic cells, and T lymphocytes express thyroid hormone receptors and respond directly to thyroid hormone signaling. Experimental studies have shown that thyroid hormones regulate neutrophil respiratory burst activity, reactive oxygen species production, macrophage phagocytic capacity, cytokine signaling, and inflammatory responses (12). In macrophages specifically, thyroid hormones have been shown to promote cell survival, as well as modulating oxidative stress pathways and influencing inflammatory signaling through thyroid hormone receptor-mediated mechanisms (14). Furthermore, thyroid hormone receptor α signaling has been implicated in the regulation of T-cell activation and regulatory T-cell function in decreasing pro-inflammatory and enhancing anti-inflammatory regulatory T cell immune responses (13).

Hypothyroidism and other thyroid disorders may therefore have important consequences for host defense against infectious diseases. Reduced thyroid hormone activity has been associated with impaired oxidative metabolism, altered leukocyte function, dysregulated cytokine production, and changes in macrophage activity that may compromise antimicrobial responses (12). Notably, many of the mentioned immune pathways influenced by thyroid hormones are also critical for controlling intracellular pathogens such as *M. tuberculosis*. Given the central role of these immune mechanisms in TB pathogenesis, thyroid dysfunction may represent an underrecognized factor contributing to altered susceptibility to infection. Therefore, understanding the metabolic and immunological consequences of thyroid hormone status provides an important foundation for examining potential links between thyroid disorders and TB susceptibility.

## 2 Nutritional Regulation of Thyroid Function

Optimal thyroid function depends on the coordinated interaction of multiple nutritional and metabolic factors. Of the nutritional component, several essential micronutrients, such as iodine, selenium, iron, zinc, and vitamin D are required for the synthesis, activation, transport, and cellular actions of thyroid hormones (15–17). These nutrients serve as structural components of thyroid hormones, cofactors for enzymatic reactions, regulators of oxidative balance, and modulators of immune function. Consequently, inadequate nutritional intake or disturbances in micronutrient homeostasis can impair thyroid hormone production and signaling, disrupt hypothalamic-pituitary-thyroid (HPT) axis regulation, and contribute to the development of thyroid dysfunction and autoimmune thyroid disease (17, 18). Given the central role of thyroid hormones in regulating metabolism, growth, and immune responses, nutritional status represents not only a critical determinant of endocrine health, but also of overall well-being.

Iodine is the principal substrate required for thyroid hormone biosynthesis and is an indispensable component of both thyroxine (T4) and triiodothyronine (T3) molecules (19). Dietary iodine is actively transported into thyroid follicular cells through the sodium-iodide symporter, oxidized by thyroid peroxidase (TPO), and incorporated into thyroglobulin during hormone synthesis (17, 19). Because the human body lacks the capacity to synthesize iodine, sufficient dietary intake is essential for maintaining normal thyroid hormone production. Although implementation of iodization programs has substantially reduced severe iodine deficiency globally, approximately 30% of the world's population remains at risk for inadequate iodine intake (19). Iodine deficiency impairs thyroid hormone synthesis, resulting in compensatory thyroid enlargement, hypothyroidism, and altered metabolic function. These observations underscore how malnutrition or undernutrition can directly influence optimal thyroid function through iodine deficiency.

Selenium is a key regulator of thyroid hormone metabolism and redox homeostasis. Notably, the thyroid gland contains the highest concentration of selenium per gram of tissue in the human body, reflecting the essential role of selenium-dependent proteins in thyroid function (20, 21). Selenium is incorporated into at least twenty-five human selenoproteins, several of which are directly involved in thyroid hormone metabolism, including iodothyronine deiodinases (DIO1, DIO2, and DIO3) that regulate the activation and inactivation of thyroid hormones. Through these enzymes, the prohormone T4 is converted into biologically active T3, allowing precise regulation of thyroid hormone action at the tissue level (22). In addition, selenium-dependent glutathione peroxidases and thioredoxin reductases protect thyroid tissue from oxidative damage generated during hormone biosynthesis (17, 20). Thyroid hormone production is an inherently oxidative process requiring hydrogen peroxide (H₂O₂) as a substrate for TPO-mediated iodination reactions. Efficient antioxidant defenses are therefore essential to prevent excessive reactive oxygen species accumulation and maintain follicular integrity. Inadequate selenium uptake through the diet can result in selenium deficiency, impairing deiodinase activity, increasing oxidative stress, disrupting thyroid hormone metabolism, and is associated with autoimmune thyroid disorders (20, 23).

Iron is another essential micronutrient involved in thyroid hormone biosynthesis. Thyroid peroxidase, the key enzyme responsible for iodide oxidation and organification, is a heme-dependent enzyme that requires adequate iron availability for optimal catalytic activity (17). Iron deficiency, the most prevalent micronutrient deficiency worldwide, may therefore impair thyroid hormone production through reduced TPO activity and altered thyroid hormone synthesis. A recent systematic review and meta-analysis demonstrated significantly lower free T3 and free T4 concentrations among individuals with iron deficiency compared with healthy controls, supporting the biological relationship between iron status and thyroid function (24). Importantly, iron deficiency frequently coexists with chronic inflammation, malnutrition, and infectious diseases, suggesting that its impact on thyroid physiology may be particularly relevant in populations at increased risk of TB.

Additional trace elements, such as zinc, contribute to thyroid homeostasis through effects on hormone synthesis, receptor signaling, and transcriptional regulation (16). Zinc participates in the structure and function of numerous transcription factors, including zinc-finger transcription factors, and is required for proper thyroid hormone receptor activity, thereby influencing the cellular response to thyroid hormones (16). Deficiency of zinc has been associated with impaired thyroid hormone metabolism, altered endocrine signaling, and immune dysfunction (16). Similarly, vitamin D has emerged as an important regulator of immune-endocrine interactions. Beyond its classical role in calcium and bone metabolism, vitamin D exerts immunomodulatory effects through regulation of T-cell differentiation, cytokine production, and immune tolerance (15, 25). Numerous observational studies have demonstrated lower circulating vitamin D concentrations in patients with hypothyroidism, Hashimoto's thyroiditis, and autoimmune thyroid disease compared with healthy controls (15). Meta-analytic evidence further suggests that vitamin D deficiency is associated with increased risk of autoimmune thyroid disorders and may correlate with disease severity and thyroid autoantibody levels (15, 25). The integrated roles of iodine, selenium, iron, zinc, and vitamin D in thyroid hormone synthesis, metabolism, antioxidant defense, and signaling are summarized in Figure 2.

**Figure 2:**
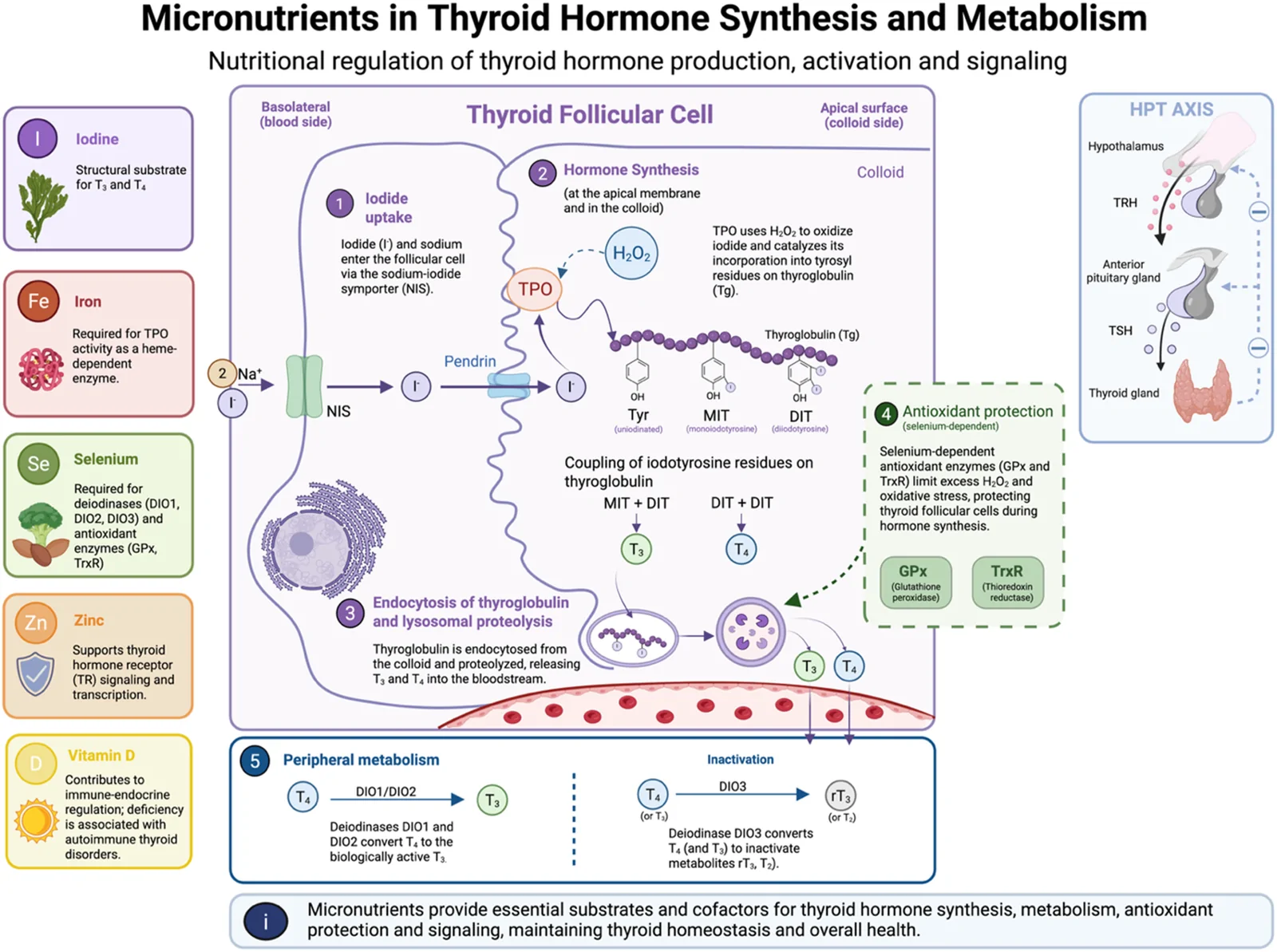
Micronutrients in thyroid hormone synthesis and metabolism. Schematic illustrating the established and proposed roles of iodine, iron, selenium, zinc, and vitamin D in thyroid hormone synthesis, antioxidant protection, metabolism and signaling. Created in https://BioRender.com.

Collectively, these findings illustrate the intricate relationship between nutrition and thyroid function. Adequate availability of iodine, selenium, iron, zinc, vitamin D, and other micronutrients is required not only for thyroid hormone synthesis and metabolism but also for maintenance of redox balance, immune regulation, and cellular homeostasis. Deficiencies in these nutrients not only contribute to endocrine dysfunction through multiple converging mechanisms, but also importantly impair and dysregulate immune responses. As discussed, many of these micronutrients are essential for effective antimicrobial immunity and regulation of inflammatory pathways. Therefore, nutritional status may represent a critical biological link connecting thyroid dysfunction, immune competence, and susceptibility to infectious diseases such as TB. The overlapping roles of key micronutrients in thyroid physiology and antimicrobial immunity are summarized in Table 1.

**Table 1:** Roles of key micronutrients in thyroid function and immunity against *M. tuberculosis.* Created in https://BioRender.com.

Nutrient	Thyroid-related role	Immune/TB-related role	Effect of deficiency	Evidence type and strength
Iodine	Required for T_3_/T_4_ synthesis	Supports immune function through normal thyroid hormone synthesis	Reduced thyroid hormone synthesis and hypothyroidism	Indirect mechanistic evidence: limited TB-specific human evidence
Selenium	Supports deiodinase activity; antioxidant protection	Supports glutathione peroxidase, redox balance, and innate/adaptive immunity	Oxidative stress, impaired T_4_ to T_3_ conversion	Primarily experimental: limited clinical evidence
Iron	Cofactor for thyroid peroxidase (TPO)	Supports macrophage and T-cell function	Reduced TPO activity, anemia, impaired immunity	Mixed clinical and mechanistic evidence: supplementation requires caution
Zinc	Supports thyroid hormone receptor signaling	T-cell development, cytokine production, antimicrobial defense	Impaired thyroid hormone signaling and immune function	Observational and indirect evidence: limited TB-specific data
Vitamin D	Immune-endocrine regulation	Supports cathelicidin production, autophagy, macrophage antimicrobial activity	Reduced antimicrobial activity	Observational association; mixed randomized-trial evidence
Glutathione	Supports redox homeostasis	Macrophage and Th1 function; reduced mycobacterial burden	Increased oxidative stress	Preliminary clinical and experimental evidence

## 3 Thyroid hormones, immune regulation, and TB susceptibility

Host protection against *M. tuberculosis* relies on the integrated actions of both innate and adaptive immune mechanisms. Following inhalation of *M. tuberculosis*, resident alveolar macrophages constitute the first line of defense and initiate a sequence of immune events that ultimately determine whether infection is contained, progresses to active disease, or establishes latent infection. Successful control of *M. tuberculosis* requires efficient macrophage activation, granuloma formation, robust T-cell responses, tightly regulated cytokine networks, and regulation of oxidative stress pathways (26, 27). As previously discussed, thyroid hormones participate in the regulation of both innate and adaptive immunity. Thyroid hormone receptors are expressed on numerous immune cell populations, including macrophages, dendritic cells, neutrophils, and T lymphocytes, enabling thyroid hormones to directly modulate immune cell differentiation, activation, and function (12, 13). Consequently, disturbances in endocrine or metabolic homeostasis that alter these immune processes may influence susceptibility to TB and disease progression.

Macrophages are central to host defense against TB because they serve as both the primary cellular target and the principal effector cell responsible for controlling mycobacterial replication. Experimental studies have demonstrated that thyroid hormones regulate macrophage survival and phagocytic activity (12, 14). Thyroid hormone signaling also influences the production of reactive oxygen species (ROS) and the expression of genes involved in antimicrobial responses (14, 27). Based primarily on mechanistic and experimental evidence, reduced thyroid hormone availability in hypothyroid states may impair macrophage activation and intracellular killing mechanisms, potentially limiting the ability of the host to restrict mycobacterial growth. Furthermore, alterations in cellular metabolism associated with hypothyroidism may affect macrophage polarization and antimicrobial function, processes increasingly recognized as critical determinants of TB outcomes (27, 28).

Adaptive immunity is equally important for TB control. Control of *M. tuberculosis* infection depends largely on CD4⁺ T lymphocytes and the generation of type 1 helper T-cell (Th1) responses characterized by the production of interferon-*γ* (IFN-*γ*) and tumor necrosis factor-α (TNF-α), cytokines that activate infected macrophages and support granuloma maintenance (26). Thyroid hormones influence T-cell development, differentiation, and activation through thyroid hormone receptor-mediated signaling pathways (13). Dysregulation of these pathways may contribute to impaired T-cell responses and altered cytokine production.

Evidence from autoimmune thyroid diseases further illustrates the close relationship between thyroid function and immune regulation. Conditions such as Hashimoto's thyroiditis and Graves' disease are characterized by lymphocytic infiltration, loss of immune tolerance, abnormal helper T-cell responses, and chronic cytokine-mediated inflammation (29, 30). Increased expression of chemokines including CXCL9, CXCL10, and CXCL11 promotes recruitment of Th1 lymphocytes and amplification of inflammatory responses within thyroid tissue, demonstrating how thyroid dysfunction can be accompanied by substantial immune dysregulation (31). Likewise, impaired suppressive activity of regulatory T cells (Tregs) has been reported in autoimmune thyroid disease, contributing to chronic inflammation and loss of self-tolerance. Because balanced Treg responses are also important for limiting immunopathology during TB infection, disturbances in immune regulation may further influence susceptibility to infection and disease progression (26, 27, 32). Similar disruptions have been observed in chronic infectious diseases, where persistent inflammation and endocrine dysregulation impair pathogen control while promoting tissue injury (27, 33).

Beyond direct effects on immune cell function, thyroid disorders may influence susceptibility to TB through broader alterations in immunometabolic regulation. Immune activation is an energetically demanding process that requires precise coordination between endocrine and immune signaling pathways. Thyroid hormones regulate cellular energy expenditure, mitochondrial function, glucose utilization, and lipid metabolism, all of which contribute to the metabolic reprogramming necessary for effective immune responses (12, 27). Chronic hypothyroidism has been associated with low-grade systemic inflammation characterized by alterations in cytokine production and immune cell metabolism (12, 34). These findings support the concept that thyroid dysfunction may affect host resistance to *M. tuberculosis* not only through direct immune mechanisms but also through broader metabolic alterations that shape immune competence.

As outlined in the preceding section, maintenance of redox homeostasis is essential for both thyroid physiology and immune function. Disruption of selenium-dependent antioxidant systems and other mechanisms regulating oxidative balance may impair immune cell function while increasing susceptibility to tissue injury during infection (17, 20, 35). Because oxidative stress also contributes to TB pathogenesis by influencing macrophage function, inflammatory responses, and bacterial clearance (27), disturbances in redox regulation may represent an additional mechanism linking thyroid dysfunction with impaired antimicrobial immunity.

Collectively, these findings suggest that thyroid dysfunction may influence TB susceptibility through multiple interconnected mechanisms involving macrophage activity, T-cell-mediated immunity, cytokine signaling, immune regulation, immunometabolic reprogramming, and oxidative stress. Although direct evidence linking hypothyroidism to TB risk remains limited, the growing recognition of immune-endocrine crosstalk supports the hypothesis that alterations in thyroid hormone signaling may affect host resistance to *M. tuberculosis*. Further investigation of these mechanisms may improve understanding of how endocrine and nutritional factors contribute to TB susceptibility and progression. Figure 3 summarizes the proposed mechanisms through which adequate and reduced thyroid hormone signaling influence immune responses against *M. tuberculosis*.

**Figure 3:**
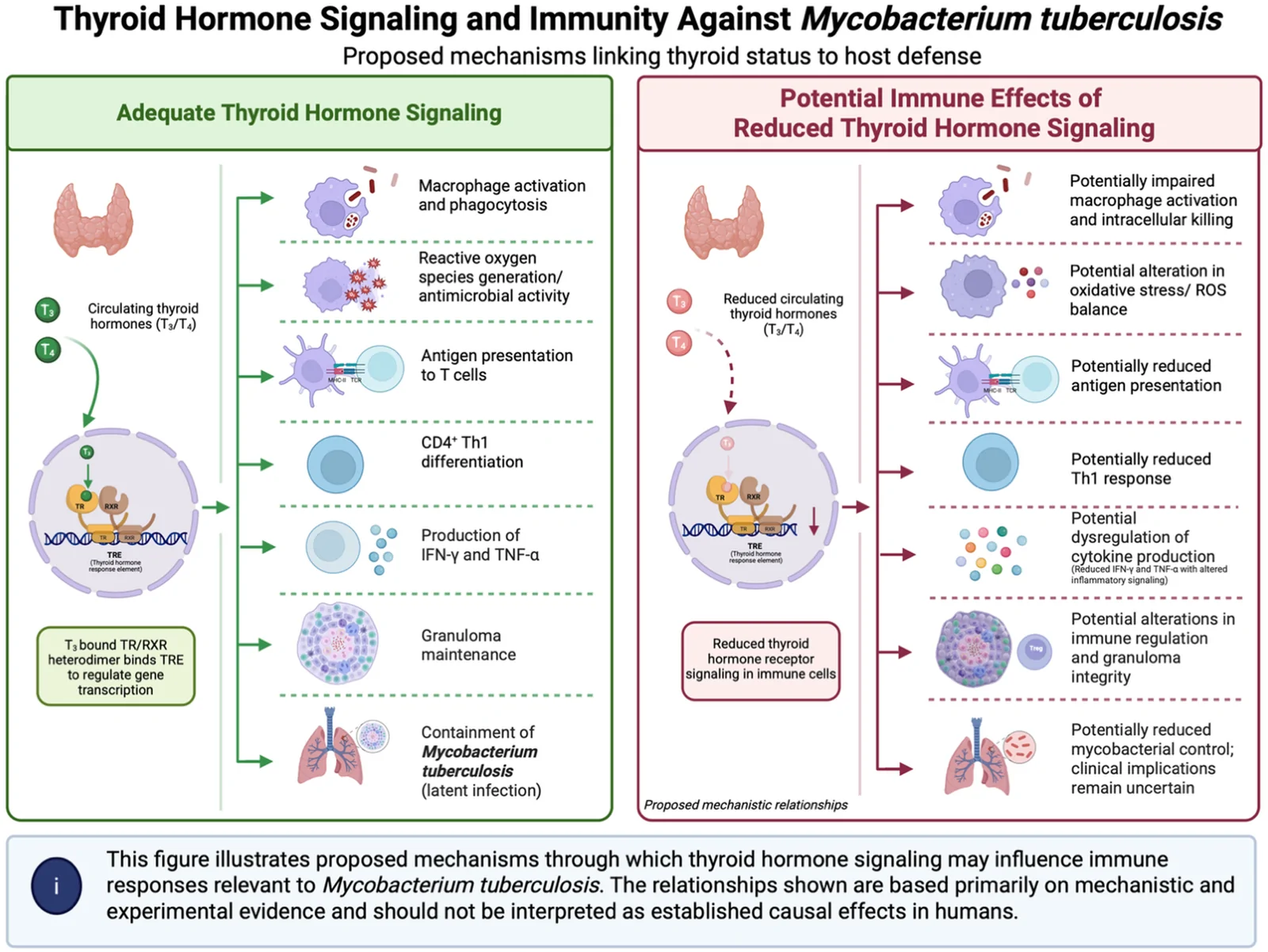
Thyroid hormone signaling and immunity against M. tuberculosis. Proposed mechanistic framework illustrating how adequate or reduced thyroid hormone signaling may influence innate and adaptive immune responses relevant to host defense against *M. tuberculosis*. The depicted relationships are supported primarily by mechanistic and experimental evidence and should not be interpreted as established causal pathways in humans. Created in https://BioRender.com.

## 4 Nutritional deficiencies and TB

Malnutrition is widely recognized as one of the strongest risk factors for TB and remains a major contributor to the global TB burden. The relationship between nutrition and TB is bidirectional: undernutrition increases susceptibility to infection and progression to active disease, while TB itself promotes nutritional deterioration through chronic inflammation, anorexia, increased energy expenditure, nutrient malabsorption, and catabolic metabolism. This interaction creates a self-perpetuating cycle in which malnutrition impairs immune function and increases vulnerability to *M. tuberculosis*, whereas active TB further worsens nutritional status and immune competence. Malnutrition profoundly weakens host defense and is often described as the leading form of acquired secondary immunodeficiency (36). Children with severe acute malnutrition are particularly vulnerable, exhibiting increased risk of TB infection, disseminated disease, and mortality compared with well-nourished children (37).

Undernutrition compromises several components of host defense that are essential for controlling *M. tuberculosis*, including macrophage function, antigen presentation, cytokine signaling, lymphocyte proliferation, and T-cell-mediated immunity (26, 27, 36). As discussed in the preceding sections, many of these pathways are also influenced by thyroid hormones and depend on adequate micronutrient availability. Consequently, nutritional deficiencies may represent an important biological link between thyroid dysfunction and increased susceptibility to TB.

Several micronutrients that are essential for thyroid physiology also contribute directly to antimicrobial immunity. Vitamin D has received particular attention because it enhances macrophage antimicrobial activity through induction of antimicrobial peptides such as cathelicidin, stimulation of autophagy, and promotion of intracellular killing of *M. tuberculosis* (38, 39). Although observational studies frequently report lower circulating vitamin D concentrations among individuals with active or latent TB (38), these associations have not consistently translated into clinical benefit from supplementation (40, 41). Recent randomized-trial meta-analyses have found no significant reduction in TB infection or progression to active disease with vitamin D supplementation and limited or inconsistent effects on treatment outcomes, including sputum smear and culture conversion (42, 43). However, potential benefits for selected clinical outcomes or patient subgroups have been reported and require further investigation (42, 43).

Similarly, zinc, selenium, and iron support both endocrine and immune function through overlapping mechanisms. Zinc is required for immune cell development, cytokine production, oxidative stress regulation, and antimicrobial defense, and deficiency has been associated with impaired T-cell responses and increased susceptibility to infectious diseases, including TB (16, 44). Selenium supports immune regulation and antioxidant defense through selenoproteins such as glutathione peroxidases and thioredoxin reductases, while experimental studies suggest that selenium deficiency impairs both innate and adaptive immunity (20, 35). Emerging research has also explored selenium nanoparticles as potential adjunctive therapies against *M. tuberculosis*, although clinical evidence remains limited (45). Iron deficiency, which frequently coexists with malnutrition and chronic inflammation, impairs macrophage function, T-lymphocyte responses, cytokine production, and antimicrobial activity (24, 36). Furthermore, inflammation-induced alterations in iron homeostasis during TB contribute to anemia of inflammation, potentially exacerbating nutritional compromise and immune dysfunction (36). Because zinc, selenium, and iron are also integral to thyroid hormone metabolism, deficiencies in these micronutrients may simultaneously impair endocrine function and host resistance to infection (16, 17, 20, 24, 35, 44).

Interpretation of micronutrient supplementation studies is limited by substantial heterogeneity across study populations and intervention protocols. Baseline nutritional status varies considerably among participants, and the effects of supplementation may differ between individuals with documented deficiency and those with adequate micronutrient status. Studies also vary in supplementation dose, formulation, duration, concomitant nutritional interventions, and timing relative to TB treatment. In addition, clinical outcomes differ across studies, ranging from biochemical and immunological markers to microbiological clearance, treatment response, and disease incidence. These methodological differences complicate direct comparisons between studies and may contribute to inconsistent findings. Consequently, evidence supporting correction of a documented nutritional deficiency should be distinguished from evidence supporting routine micronutrient supplementation specifically for TB prevention or treatment.

Altered redox homeostasis represents another shared mechanism linking nutritional deficiency with TB susceptibility. During infection, macrophages generate ROS as part of the antimicrobial response; however, excessive oxidative stress may promote tissue injury and impair immune function (46, 47). Patients with TB frequently exhibit depleted glutathione (GSH) levels, compromising antioxidant defenses, macrophage function, cytokine regulation, and T-cell-mediated immunity (48). Experimental and clinical studies suggest that GSH supplementation may reduce oxidative stress, enhance Th1-associated immune responses, increase production of IFN-*γ* and TNF-α, and decrease intracellular mycobacterial burden (48, 49). Given the close relationship between GSH metabolism and selenium-dependent antioxidant systems, these findings further illustrate the interconnected roles of nutrition, redox balance, and immune competence in TB pathogenesis.

Collectively, these findings suggest that nutritional deficiencies may influence TB-related immune responses through multiple mechanisms involving altered cytokine signaling, oxidative stress, and disruption of antimicrobial defense pathways. Micronutrients such as vitamin D, zinc, selenium, iron, and GSH not only support immune function but also contribute to thyroid homeostasis, suggesting substantial biological overlap between nutritional status, endocrine regulation, and host resistance to *M. tuberculosis*. Several of these micronutrients participate in both thyroid hormone regulation and antimicrobial immunity, providing a biologically plausible, although not yet clinically established, link between nutritional deficiency, endocrine dysfunction, and TB susceptibility.

## 5 Connecting nutrition, thyroid dysfunction, and TB susceptibility

The evidence presented throughout this review suggests that nutrition, thyroid function, and susceptibility to TB are interconnected through a complex network of endocrine, metabolic, and immunological pathways. Rather than acting as independent risk factors, nutritional status and thyroid health converge on many of the biological processes that determine host resistance to *M. tuberculosis*. Nutritional deficiencies can impair thyroid hormone synthesis and metabolism, while thyroid dysfunction may alter immune responses involved in mycobacterial control. Together, these observations provide a biologically plausible framework through which nutritional and endocrine disturbances could influence TB susceptibility, although direct clinical evidence remains limited.

The strength of evidence supporting the individual components of this framework varies considerably. Human epidemiological evidence strongly supports undernutrition as a risk factor for TB, while human studies also demonstrate associations between specific micronutrient deficiencies and altered thyroid function. In contrast, direct human evidence linking thyroid dysfunction itself to increased TB susceptibility remains limited. Although population-based observational data have identified an association between hypothyroidism and TB, such findings cannot establish causality and may be influenced by confounding factors, including nutritional status, comorbidities, socioeconomic conditions, chronic inflammation, and medication exposure. Much of the proposed connection between thyroid dysfunction and impaired antimycobacterial immunity therefore remains based on mechanistic and experimental evidence rather than prospective clinical studies. Accordingly, the integrated nutritional–endocrine–immune framework presented here should be considered biologically plausible and hypothesis-generating rather than an established causal pathway.

Nutrition is a fundamental determinant of thyroid homeostasis, with adequate intake of iodine, selenium, iron, zinc, and vitamin D required to maintain normal thyroid hormone synthesis, metabolism, and signaling. Deficiencies in these micronutrients can disrupt thyroid physiology, impair hypothalamic-pituitary-thyroid (HPT) axis regulation, and increase the risk of hypothyroidism or autoimmune thyroid disease (15, 17, 20, 24). Importantly, deficiencies of these same nutrients remain highly prevalent in many regions where TB is endemic, particularly in low- and middle-income countries affected by food insecurity, undernutrition, and chronic infectious disease burdens (36). Consequently, inadequate micronutrient status may represent an upstream factor that simultaneously impairs endocrine function and weakens host immune defenses before exposure to *M. tuberculosis* even occurs.

The influence of nutrition extends beyond thyroid hormone production because thyroid hormones regulate multiple immune pathways essential for antimicrobial defense. Through interactions with thyroid hormone receptors expressed on macrophages, dendritic cells, neutrophils, and lymphocytes, thyroid hormones influence macrophage activation, T-cell differentiation, cytokine production, and immunometabolic pathways required for controlling *M. tuberculosis* (12–14, 27). Consequently, nutritional deficiencies that affect thyroid hormone availability could plausibly influence host immune responses relevant to TB. Although direct epidemiological evidence remains limited, experimental and mechanistic evidence supports further investigation of immune-endocrine crosstalk as a potential link between thyroid dysfunction and susceptibility to mycobacterial infection (33).

Several micronutrients occupy key positions at the intersection of thyroid physiology and antimicrobial immunity. Selenium, iron, zinc, and vitamin D contribute not only to thyroid hormone regulation but also to macrophage function, T-cell responses, cytokine signaling, antioxidant defense, and intracellular antimicrobial activity (15–17, 20, 24, 35, 38, 39, 44). Consequently, deficiencies in these nutrients may exert compounded effects across endocrine and immune systems, amplifying susceptibility to infection.

Disturbances in redox homeostasis may represent an additional mechanism linking nutrition, thyroid dysfunction, and TB susceptibility. As discussed previously, selenium-dependent antioxidant enzymes and GSH are essential for maintaining both thyroid function and effective antimicrobial immunity (17, 20, 35). Disruption of these antioxidant systems may therefore impair thyroid physiology while reducing the capacity of immune cells to control *M. tuberculosis*, contributing to inflammation, tissue injury, and disease progression (46, 48).

The relationship between thyroid dysfunction and TB is likely bidirectional. Chronic infection and systemic inflammation alter HPT axis regulation through the actions of pro-inflammatory cytokines such as IL-1, IL-6, and TNF-α, thereby influencing thyroid hormone production and metabolism (33). Furthermore, antitubercular agents including ethionamide, prothionamide, and para-aminosalicylic acid have been associated with inhibition of thyroid peroxidase activity and disruption of iodine organification, resulting in hypothyroidism in approximately 17% of patients receiving treatment for multidrug-resistant TB (50). These observations suggest that thyroid dysfunction may arise not only as a contributor to impaired immune defense but also as a consequence of chronic infection and its treatment.

Taken together, the available evidence supports a conceptual framework in which nutritional deficiencies contribute to thyroid dysfunction, thyroid dysfunction alters immune and metabolic pathways required for protection against *M. tuberculosis*, and TB infection further exacerbates nutritional and endocrine disturbances. Within this model, undernutrition serves as an initiating factor that disrupts both endocrine and immune homeostasis, creating conditions favorable for infection. Once infection develops, chronic inflammation, increased metabolic demands, nutrient depletion, and treatment-related endocrine complications may further worsen thyroid function and nutritional status, creating a self-reinforcing cycle that contributes to increased susceptibility to TB and poorer clinical outcomes.

Although direct clinical evidence linking thyroid disorders to TB risk remains limited, the convergence of nutritional, endocrine, immunological, and metabolic pathways provides a biologically plausible rationale for further investigation. Future studies should determine whether optimization of nutritional status, targeted micronutrient supplementation, and early identification and management of thyroid dysfunction can improve immune competence and reduce TB susceptibility in high-risk populations. A greater understanding of these interactions may also facilitate the development of integrated nutritional and endocrine interventions as adjunctive strategies for TB prevention, treatment, and long-term management.

## 6 Potential nutritional and therapeutic interventions

The growing recognition of interactions among nutritional status, thyroid function, and immune regulation suggests that nutritional interventions may represent a potential adjunctive approach for addressing nutritional deficiencies and supporting immune and endocrine function in individuals at risk for or affected by TB. Although antimicrobial therapy remains the cornerstone of TB treatment, increasing evidence supports the importance of addressing nutritional deficiencies and metabolic disturbances as complementary components of disease management. Given that many micronutrients influence both thyroid physiology and antimicrobial immunity, nutritional optimization may provide benefits that extend beyond correction of isolated deficiencies.

Dietary support and nutritional rehabilitation are particularly important in populations affected by undernutrition, where nutritional deficiencies frequently coexist with increased TB risk and poorer treatment outcomes (36). Adequate intake of calories, protein, vitamins, and trace elements is necessary to support immune cell proliferation, cytokine production, tissue repair, and recovery from infection. Nutritional rehabilitation has been associated with improvements in body composition, immune function, and treatment response among patients with active TB, highlighting the importance of integrating nutritional assessment into routine clinical care (36). Furthermore, because undernutrition contributes to both endocrine dysfunction and impaired immunity, dietary interventions may simultaneously support thyroid health and host defense mechanisms.

Targeted micronutrient supplementation has also received considerable attention as a potential adjunctive strategy for improving immune competence and reducing TB susceptibility. Vitamin D is among the most extensively studied micronutrients in this context due to its ability to enhance macrophage antimicrobial activity, induce production of antimicrobial peptides such as cathelicidin, and modulate inflammatory responses (38, 39). Although observational studies demonstrate associations between vitamin D deficiency and increased TB risk, recent randomized-trial meta-analyses have not demonstrated a consistent benefit of vitamin D supplementation for preventing TB infection or progression to active disease (42, 43). Similarly, supplementation during TB treatment has generally not produced consistent improvements in major microbiological outcomes, although benefits have been reported for selected secondary outcomes and patient subgroups (42, 43). Therefore, current evidence does not support routine vitamin D supplementation specifically for TB prevention or as adjunctive TB therapy in unselected populations. Further studies are needed to determine whether individuals with documented vitamin D deficiency or other specific subgroups may derive greater benefit.

Selenium supplementation has attracted interest because of its dual roles in thyroid hormone metabolism and antioxidant defense. Selenium-dependent enzymes regulate thyroid hormone activation while simultaneously protecting tissues from oxidative damage and supporting immune function (20, 35). Some studies have reported reductions in thyroid autoantibody concentrations and changes in markers of thyroid function following selenium supplementation in autoimmune thyroid disease; however, the clinical significance and generalizability of these findings remain uncertain (20). Emerging experimental evidence also suggests that selenium-based therapies may possess antimicrobial and immunomodulatory properties relevant to TB pathogenesis (45). While current evidence remains insufficient to support routine selenium supplementation specifically for TB prevention, maintenance of adequate selenium status may contribute to both endocrine and immune homeostasis.

Similarly, zinc and iron supplementation may offer potential benefits in selected populations with documented deficiencies. Zinc plays a critical role in immune cell development, cytokine production, and thyroid hormone signaling, while iron is essential for thyroid hormone synthesis, oxygen transport, cellular metabolism, and antimicrobial immune responses (16, 17, 24, 44). Because deficiencies of these micronutrients are common in resource-limited settings and among individuals with chronic infectious diseases, correction of documented deficiencies may support normal thyroid and immune function; however, evidence that supplementation independently improves TB-specific clinical outcomes remains limited. Supplementation strategies should be carefully tailored to individual nutritional needs, as excessive intake of certain micronutrients may also have adverse effects on endocrine or immune function.

Antioxidant-based interventions represent another promising therapeutic avenue. Oxidative stress contributes to both thyroid dysfunction and TB pathogenesis, suggesting that restoration of redox balance suggesting that restoration of redox balance could represent a potential therapeutic target (20, 46). GSH, the body's principal intracellular antioxidant, has emerged as a particularly attractive candidate due to its central role in regulating oxidative stress, cytokine production, macrophage activity, and T-cell function (48). Studies have demonstrated that patients with TB frequently exhibit depleted glutathione levels, and experimental investigations suggest that GSH supplementation may enhance antimycobacterial immune responses, reduce oxidative stress, increase production of protective cytokines such as IFN-*γ* and TNF-α, and decrease intracellular mycobacterial burden (48, 49). Because glutathione metabolism is closely linked to selenium-dependent antioxidant systems, strategies aimed at improving antioxidant capacity may provide synergistic benefits for both thyroid function and host defense.

Given the interconnected nature of nutritional status, endocrine regulation, and immunity, future therapeutic approaches may benefit from a more integrated framework that extends beyond pathogen-directed treatment alone. Routine nutritional screening, assessment of micronutrient status, and monitoring of thyroid function may help identify individuals at increased risk for immune dysfunction and adverse TB outcomes. Particular attention may be warranted in populations affected by malnutrition, autoimmune thyroid disease, multidrug-resistant TB, or prolonged antimicrobial therapy, where nutritional and endocrine disturbances are more likely to occur (36, 50). Although current evidence supports strong biological links among nutrition, thyroid dysfunction, and TB susceptibility, prospective clinical studies are needed to determine whether targeted nutritional interventions and thyroid-directed management strategies can meaningfully reduce TB risk or improve treatment outcomes. Potential nutritional and clinical interventions, along with their proposed benefits and current limitations are summarized in Table 2.

**Table 2:** Potential nutritional and clinical interventions to support thyroid function and immunity in tuberculosis. Created in https://BioRender.com.

Intervention	Primary target	Proposed thyroid benefit	Proposed TB/immune benefit	Evidence and limitations
Nutritional rehabilitation	Undernutrition	May support thyroid and metabolic homeostasis	May support immune recovery and tissue repair	Moderate clinical evidence; protocols vary
Vitamin D correction	Vitamin D deficiency	May support thyroid-immune regulation	Cathelicidin, autophagy, macrophage activation	Mixed clinical trial evidence
Selenium supplementation	Selenium deficiency	Supports deiodinases and antioxidant enzymes	May support immune and redox function	Limited clinical evidence for TB use
Zinc supplementation	Zinc deficiency	Supports receptor signaling	May support T-cell and cytokine responses, particularly in deficiency	Limited evidence; benefit may depend on deficiency
Iron correction	Iron deficiency	May restore TPO cofactor availability in documented iron deficiency	May support oxygen transport and immune function	Indirect evidence; infection-related risks
Glutathione-based therapy	Oxidative stress	May support systemic redox balance	May increase IFN-*γ* andTNF-α and reduce intracellular mycobacterial burden	Preliminary clinical and experimental evidence
Thyroid screening and treatment	Thyroid dysfunction	Enables early identification and management of hypothyroidism	May indirectly improve immune competence	Insufficient evidence for TB outcomes

Ultimately, optimization of nutritional status represents an important component of general health and may support normal immune and thyroid function, particularly in populations affected by undernutrition. Whether specific nutritional interventions improve resistance to TB or alter TB-related clinical outcomes requires further investigation. Continued investigation of nutritional, endocrine, and immunological interactions may facilitate development of novel adjunctive therapies that complement existing TB control strategies and improve long-term health outcomes in vulnerable populations.

## 7 Knowledge gaps and future research priorities

Despite increasing recognition of interactions among nutritional status, thyroid function, and immune regulation, important gaps remain in understanding their relevance to TB susceptibility. Direct prospective human evidence linking thyroid dysfunction to the development of TB is limited, and much of the current biological framework is derived from observational associations and mechanistic studies. Future prospective cohort studies should determine whether thyroid dysfunction independently predicts TB infection or progression to active disease after accounting for potential confounding factors such as nutritional status, comorbidities, socioeconomic conditions, and chronic inflammation. Studies should also evaluate whether specific micronutrient deficiencies modify the relationship between thyroid function and TB susceptibility and identify whether patient populations are at greater risk. In addition, adequately powered clinical trials are needed to determine whether correction of documented micronutrient deficiencies or management of thyroid dysfunction meaningfully affects TB-related clinical outcomes. Standardization of baseline nutritional assessment, supplementation dose and duration, thyroid function measurements, and clinical outcomes would improve comparability across studies. Collectively, these approaches will be necessary to determine whether the proposed nutritional–endocrine–immune relationships have clinical significance and can ultimately inform evidence-based screening or therapeutic strategies.

## 8 Conclusion

TB remains a major global health challenge, particularly in regions where undernutrition and micronutrient deficiencies are highly prevalent. Emerging evidence suggests that nutritional status, thyroid function, and immune competence are closely interconnected and may collectively influence susceptibility to *M. tuberculosis*. Adequate availability of essential micronutrients, including iodine, selenium, iron, zinc, and vitamin D, is necessary for maintaining thyroid hormone synthesis, metabolic homeostasis, and effective immune responses. Deficiencies in these nutrients may contribute to thyroid dysfunction, impair immune regulation, disrupt antioxidant defenses, and compromise the host's ability to control mycobacterial infection. Although direct evidence linking hypothyroidism to TB susceptibility remains limited, current findings support a biologically plausible relationship mediated through shared endocrine, metabolic, and immunological pathways.

This review highlights a conceptual framework in which nutritional deficiencies may contribute to thyroid dysfunction, thyroid dysfunction may alter immune responses critical for protection against *M. tuberculosis*, and TB infection may further exacerbate nutritional and endocrine disturbances. These interactions may create a self-perpetuating cycle that increases vulnerability to infection and worsens clinical outcomes. Given the substantial overlap between pathways involved in thyroid regulation and antimicrobial immunity, nutritional optimization and early recognition of thyroid dysfunction warrant further investigation as potential adjunctive approaches for supporting host defense. However, much of the current evidence is derived from observational associations and mechanistic studies, and direct clinical evidence establishing thyroid dysfunction as an independent determinant of TB susceptibility remains limited. Accordingly, current evidence does not support routine thyroid screening solely for the purpose of reducing TB risk. Further prospective and interventional studies are required before the proposed nutritional and thyroid-directed approaches can be translated into TB-specific clinical recommendations.
